# Evaluation of the Anti-Inflammatory and Chondroprotective Effect of Celecoxib on Cartilage *Ex Vivo* and in a Rat Osteoarthritis Model

**DOI:** 10.1177/19476035221115541

**Published:** 2022-08-05

**Authors:** Mirella J.J. Haartmans, Ufuk Tan Timur, Kaj S. Emanuel, Marjolein M.J. Caron, Ralph M. Jeuken, Tim J.M. Welting, Gerjo J.V.M. van Osch, Ron M.A. Heeren, Berta Cillero-Pastor, Pieter J. Emans

**Affiliations:** 1 Laboratory for Experimental Orthopedics, Department of Orthopaedic Surgery, Maastricht University, Maastricht, The Netherlands; 2 Maastricht MultiModal Molecular Imaging Institute (M4i), Division of Imaging Mass Spectrometry, Maastricht University, Maastricht, The Netherlands; 3 Department of Orthopaedic Surgery, Amsterdam Movement Sciences, Amsterdam UMC, University of Amsterdam, Amsterdam, The Netherlands; 4 Department of Orthopaedics and Sports Medicine, Erasmus MC, University Medical Center Rotterdam, Rotterdam, The Netherlands; 5 Department of Otorhinolaryngology, Erasmus MC, University Medical Center Rotterdam, Rotterdam, The Netherlands; 6 MERLN Institute for Technology-Inspired Regenerative Medicine, Department of Cell Biology-Inspired Tissue Engineering, Maastricht University, Maastricht, The Netherlands; 7 Laboratory for Experimental Orthopedics, Joint Preserving Clinic, Department of Orthopaedic Surgery, Maastricht University Medical Centre+, Maastricht, The Netherlands

**Keywords:** knee osteoarthritis, NSAIDs, prostanoid, joint inflammation, proteomics

## Abstract

**Objective:**

The potential chondroprotective effect of celecoxib, a nonsteroidal anti-inflammatory drug and selective cyclooxygenase-2 inhibitor used to reduce pain and inflammation in knee osteoarthritis patients, is disputed. This study aimed at investigating the chondroprotective effects of celecoxib on (1) human articular cartilage explants and (2) in an *in vivo* osteoarthritis rat model.

**Design:**

Articular cartilage explants from 16 osteoarthritis patients were cultured for 24 hours with celecoxib or vehicle. Secreted prostaglandins (prostaglandin E_2_, prostaglandin F_2α_, prostaglandin D_2_) and thromboxane B2 (TXB2) concentrations were determined in medium by ELISA, and protein regulation was measured with label-free proteomics. Cartilage samples from 7 of these patients were analyzed for gene expression using real-time quantitative polymerase chain reaction. To investigate the chondroprotective effect of celecoxib *in vivo*, 14 rats received an intra-articular injection of celecoxib or 0.9% NaCl after osteoarthritis induction by anterior cruciate ligament transection and partial medial meniscectomy (ACLT/pMMx model). Histopathological scoring was used to evaluate osteoarthritis severity 12 weeks after injection.

**Results:**

Secretion of prostaglandins, target of Nesh-SH3 (ABI3BP), and osteonectin proteins decreased, whereas tissue inhibitor of metalloproteinase 2 (TIMP-2) increased significantly after celecoxib treatment in the human (*ex vivo*) explant culture. Gene expression of a disintegrin and metalloproteinase with thrombospondin motifs 4 and 5 (ADAMTS4/5) and metalloproteinase 13 (MMP13) was significantly reduced after celecoxib treatment in human cartilage explants. Cartilage degeneration was reduced significantly in an *in vivo* osteoarthritis knee rat model.

**Conclusions:**

Our data demonstrated that celecoxib acts chondroprotective on cartilage *ex vivo* and a single intra-articular bolus injection has a chondroprotective effect *in vivo*.

## Introduction

Knee osteoarthritis (OA) patients suffer from joint pain and immobility, which is usually treated by nonsteroidal anti-inflammatory drugs (NSAIDs) and physical therapy, with total knee arthroplasty (TKA) as an end-stage disease solution.^1,2^ Over the years, several disease-modifying OA drugs came to attention.^3^ Selective cyclooxygenase-2 (COX-2) inhibitors are a type of disease-modifying OA drugs and are investigated for their effect on the inhibition of prostaglandins (PGs). PGs, secreted by intra-articular tissues, are an important class of signaling molecules present in synovial fluid and involved in inflammation.^4,5^

The family of eicosanoids consists of 5 different subtypes: prostaglandin E_2_ (PGE_2_), prostaglandin D_2_ (PGD_2_), prostaglandin I_2_ (PGI_2_), prostaglandin F_2α_ (PGF_2α_), and thromboxane A_2_ (TXA_2_).^6^ Inhibition of their production has been shown to provide pain-reducing effects.^6^ It has been suggested that PGE_2_ acts catabolic and induces cartilage degradation by inhibiting proteoglycan synthesis and stimulating matrix degradation.^7,8^ Studies on the actions of prostanoids on articular tissues have focused mainly on cartilage and PGE_2_, proposing it as a catabolic or anti-anabolic factor,^7-9^ while also anti-catabolic effects on chondrocytes have been reported.^10-12^

An effective way to target prostanoid synthesis is by blocking COX activity.^6^ At least 2 COX isoforms have been described, COX-1 and COX-2, the latter being considered to be associated with inflammation.^13^ COX-2 inhibitors have been designed to target the inflammatory COX-2 while circumventing inhibition of COX-1.^13^ An example of a specific COX-2 inhibitor is celecoxib, which is currently used as an analgesic by patients with inflammatory arthritic diseases.^14^ Besides being a drug with analgesic properties, evidence on the OA disease-modifying effects of celecoxib is now increasing, with *ex vivo* and *in vivo* data showing chondroprotective effects,^15-20^ including beneficial effects on cartilage matrix turnover and suppression of proinflammatory factors. However, other studies report contradictory data showing the absence of a chondroprotective effect of celecoxib in a groove OA-model in dogs,^21^ in human patients with knee OA,^22^ or in a ligament transection and meniscectomy mouse model.^23^ This may be related to differences in OA severity, timing of administration after OA induction, or lower local concentrations of celecoxib in the knee joint due to oral administration and insufficient patient compliance.^24^

Whereas oral administration of celecoxib can be accompanied by negative side effects such as cardiovascular disease,^20^ the effect of intra-articular administration, which has not been used in clinic yet, has been studied briefly.^18,25-27^ Whereas some studies report improvement in cartilage degeneration scores,^18,25^ others do not report improvement.^26,27^ Differences in study design, including dosage, timing of treatment, or way of administration (with or without the use of dose delivery systems), and use of scoring system might be related to the outcome of results.

Due to the controversy of previous literature and to further clarify the chondroprotective effect of celecoxib, this work aims at studying the effect of celecoxib *in vitro* and *in vivo*. In the current study, we aimed at investigating (1) the *ex vivo* biomolecular mechanism and chondroprotective effect of celecoxib in cartilage biopsies by measuring (a) prostaglandin and protein secretion in the culture medium and (b) gene expression in the cartilage explants. In addition, (2) the *in vivo* effect of an intra-articular celecoxib injection on cartilage was investigated in an OA-induced rat model.

We hypothesize that celecoxib acts chondroprotective by reducing the release of inflammatory prostaglandins and that it might act as an anti-proteolytic drug by reducing the gene expression of specific proteolytic enzymes (such as metalloproteinases [MMPs])^28^ and altering secreted proteins. To test this hypothesis, we analyzed the prostaglandin release by cartilage explants treated with celecoxib or dimethylsulfoxide (DMSO) and performed gene expression analysis of important proteolytic enzymes known to be involved in knee OA pathophysiology. Subsequently, we analyzed the protein secretome in cartilage-conditioned medium.

We expect that the intra-articular injection of celecoxib has a chondroprotective effect and that a local administration would have a larger effect compared with oral administration. Thus, we investigated the chondroprotective capacity of a single intra-articular bolus injection of celecoxib in an *in vivo* surgically induced rat OA model.

## Method

### *Ex Vivo* Study

#### Human tissue explant cultures

From 16 human subjects with knee OA who underwent TKA, full-thickness cartilage explants were obtained (MEC approval 11-4-040). Cartilage pieces from femoral condyles and tibia plateaus were cut into small pieces, washed thoroughly with 0.9% NaCl 3 times, and cultured at 37 °C and 5% CO_2_ in suspension for 24 hours with a concentration of 100 mg tissue/ml in Dulbecco’s modified Eagle’s medium (DMEM-F12 low glucose; Invitrogen, Carlsbad, CA) supplemented with 1% insulin-transferrin-selenite media supplement (ITS) (Invitrogen) and 1% antibiotic/antimycotic (Invitrogen).^29^ In addition, celecoxib (LC Laboratories, Woburn, MA) was dissolved in DMSO (vehicle, DMSO; Sigma-Aldrich, St Louis, MO) and added to the culture medium in a 10 µM final concentration. The concentration of celecoxib was determined based on earlier dose-response experiments.^19,29^ DMSO was added 1:1,000 to cultures without celecoxib as a control. After 24 hours, cartilage-conditioned medium was harvested, centrifuged at 1200 RPM for 8 minutes, and the supernatant was frozen at −80 °C. Media were stored for a maximum of 4 weeks before prostanoid analysis. After 24 hours of culture, tissue explants were snap-frozen in liquid nitrogen and stored at −80 °C until being processed for RNA isolation.

#### Prostanoid measurement in conditioned media

PGE_2_, PGF_2α_, PGD_2_, and TXB_2_ (a stable metabolite of TXA_2_) concentrations were determined in OA cartilage-conditioned medium by a competitive enzyme-linked immunosorbent assay (ELISA) according to the manufacturer’s instructions (Cayman Chemicals, Ann Arbor, MI). ELISA for PGI_2_ was not performed in these experiments, as PGI_2_ is unstable in culture medium and previous experiments showed that it could not be measured reliably. PG concentrations in the samples were calculated from a calibration curve using standards supplied by the manufacturer.

#### Gene expression analysis: RNA isolation and RT-qPCR

Frozen cartilage samples from 7 patients were homogenized with a Mikro-Dismembrator S (B. Braun Biotech International GmbH, Melsungen, Germany) and suspended in 1 ml TRIzol (Thermo Fisher Scientific, Waltham, MA)/100 mg tissue. RNA extraction, purification, and quantification have been published earlier, as was the complementary DNA (cDNA) synthesis using both commercially available Rneasy Micro Kit (QIAGEN, Hilden, Germany) and Eurogentec kits (Eurogentec, Seraing, Belgium).^29^ Gene expression was analyzed using quantitative real-time polymerase chain reaction (RT-qPCR) as described earlier.^29^ Validated primer sequences of *28S* ribosomal RNA (rRNA), *PPIA* (peptidylprolyl isomerase A), *GAPDH* (glyceraldehyde 3-phosphate dehydrogenase), *MMP13* (matrix metalloproteinase 13), *COL2A1* (collagen type 2 alpha 1), *ACAN* (aggrecan), *ADAMTS4* (a disintegrin and metalloproteinase with thrombospondin motifs 4), and *ADAMTS5* (a disintegrin and metalloproteinase with thrombospondin motifs 5) were published earlier.^29^ Amplification efficiencies of the primers were between 0.9 and 1.05. Gene expression analysis was performed based on normalization to the best housekeeper index, based on gene expression levels of *28S, PPIA*, and *GAPDH*, as previously described.^30^ These housekeeping genes were selected before as reliable.^29^

#### Mass spectrometry analysis of conditioned media

An untargeted proteome analysis on OA cartilage-conditioned medium with and without celecoxib treatment was performed. For protein precipitation, the samples were centrifuged for 5 minutes at 1,200 RPM to remove potential cells and for 10 minutes at 13,000 RPM to remove potential macromolecular aggregates. Subsequently, 10 μl of 0.2% sodium deoxycholate (DOC; Sigma-Aldrich) was added to each sample, vortexed, and incubated for 10 minutes at 4 °C. Then, 10 μl of trichloroacetic acid (TCA; Sigma-Aldrich) was added, vortexed, and incubated for 1 hour at 4 °C. The samples were centrifuged for 10 minutes at 13,000 RPM and the supernatant was removed. The remaining pellet was washed with 1 ml of cold acetone (−20 °C) 3 times with intermediate centrifugation (13,000 RPM for 10 min) and removal of the supernatant. Dried protein pellets were dissolved in 50 μl of 5 M urea (GE Healthcare, Chicago, IL)/50 mM ammonium bicarbonate (Sigma-Aldrich) sample buffer and stored at −20 °C until further processing. The protein content of each sample was measured using Bradford Protein Assay (Bio-Rad Laboratories, Hercules, CA) according to the manufacturer’s protocol. Absorption was determined at 595 nm (optical density). A total of 6.6 µg protein per sample was used for protein digestion. Five microliters of 20 mM dithiothreitol (Sigma-Aldrich) in ULC/MS grade water (Biosolve, Valkenswaard, the Netherlands) was added to each sample, vortexed, and incubated for 45 minutes at room temperature. Subsequently, 6 μl of 40 mM iodoacetamide (Sigma-Aldrich) in ULC/MS grade water (Biosolve) was added, vortexed, and incubated for 45 minutes at room temperature in the dark. Ten microliters of 20 mM dithiothreitol (Sigma-Aldrich) was added to the solution, vortexed, and incubated at room temperature for 45 minutes to stop the reaction. A trypsin/Lys-C solution in resuspension buffer (Promega, Leiden, the Netherlands) was added in an enzyme to protein ratio of 1:25. The samples were vortexed and incubated in a water bath (37 °C) for 2 hours, before spinning the samples down and adding 200 μl of 50 mM ammonium bicarbonate (Sigma-Aldrich) sample buffer. Again, samples were vortexed and incubated in a water bath (37 °C) overnight.

Finally, 30 μl of 20% acetonitrile (ACN) (Biosolve)/10% formic acid (FA) (Biosolve) was added and samples were vortexed to stop the reaction. Samples were centrifuged for 30 minutes at 13,000 RPM to remove possible particles and were stored at −20 °C until liquid chromatography-tandem mass spectrometry (LC-MS/MS).

Proteomic analysis was performed on a Thermo Scientific Ultimate 3000 Rapid Separation UHPLC system (Dionex, Amsterdam, the Netherlands), coupled to a Q-Exactive HF mass spectrometer (Thermo Fisher Scientific). The UHPLC system was equipped with a PepSep C18 analytical column (15 cm, ID 75 µm, 1.9 µm Reprosil, 120Å). Samples were desalted on an online installed C18 trapping column and then separated on an analytical column with a 90-minute linear gradient (5%-35% ACN with 0.1% FA, flow rate 300 nl/min). The scans were performed in data-dependent acquisition mode (DDA). Full mass spectrometry (MS) scans were executed from 250 to 1250 *m/z* (mass to charge ratio) at a resolution of 120,000, followed by tandem MS (MS/MS) scans on the 15 most intense ions at a resolution of 15,000.^31^

### *In Vivo* Study

#### Surgery for induction of osteoarthritis

The chondroprotective effect of celecoxib was investigated in a rat OA model, anterior cruciate ligament (ACL) transection in combination with a partial medial meniscectomy (ACLT/pMMx). The primary outcome measure was the cartilage degeneration score according to the Osteoarthritis Research Society International (OARSI) histopathology initiative for the rat.^32^ The study was performed in accordance with the ARRIVE (Animal Research: Reporting of *In Vivo* Experiments) guidelines.^33^ All animal experimental protocols were approved by the Maastricht University Animal Ethics Committee (DEC13-052).

Sample size was calculated according to the formula of L. Sachs^34^ with α = 0.05, power = 0.80 (β = 0.20), spread of σ = 20%, and an effect size of δ = 33%,^35^ resulting in *n* = 6 knees per group. We expected a 10% dropout for all groups. Therefore, we included 7 knees per group.

Fourteen 3-month-old, male Lewis rats (Charles River, ‘s-Hertogenbosch, the Netherlands) were allowed to acclimatize for 1 week, before the start of the experiments. Animals were housed in pairs, kept on a 12-hour dark/light cycle, and fed *ad libitum*. OA was surgically induced in the right knee by the ACLT/pMMx OA model.^32^ The left knees were used as healthy controls. Only male rats were included in this study to prevent an influence of hormonal fluctuation during menstrual cycle of female rats.

In brief, rats were anesthetized in a chamber containing 1% isoflurane (Isoflo; Abbott Laboratories, Chicago, IL). The right knee joint of each rat was shaved, cleaned, and disinfected with iodine (Eurovet Animal Health, Bladel, the Netherlands). The skin was incised with a longitudinal incision on the medial side of the joint. Then, the joint capsule was incised on the medial side of the patellar tendon, which provided access to the joint space. The patella was dislocated laterally and the ACL was transected using a surgical blade (size 11). Transection was confirmed by a manually performed anterior drawer test. In addition, the anterior part of the medial meniscus was removed using a surgical scissor. The joint capsule and skin were closed with Vycril 4-0 sutures. Left knees were kept intact.

Animals were allowed to move freely in their cage and were checked daily for general health and experiment-related discomfort for 3 weeks. Four weeks after surgery, rats were randomly assigned to 2 experimental groups using the block randomization method. The treatment group received an intra-articular injection of 25 μl of 0.9% NaCl containing 92.25 ng of celecoxib in both the operated (group 1a: OA celecoxib) and nonoperated leg (group 1b: healthy celecoxib). The control group received an intra-articular injection of 25 μl of 0.9% NaCl in both legs (group 2a and group 2b, respectively: OA control and healthy control). This amount of celecoxib was based on previous literature.^19,29^ Twelve weeks after injection, rats were anesthetized with 1% isoflurane and killed by cervical dislocation. OA severity was assessed by scoring histological sections of rat knee joints using the OARSI histopathology initiative for the rat by 2 blinded observers.^32^

#### Tissue preparation and histology

Rat knee joints were carefully resected and fixed with 3.7% paraformaldehyde (VWR International, Radnor, PA) in 0.1 M phosphate-buffered saline at 4 °C for 1 week. Next, tissues were decalcified in 0.5 M ethylenediamenetetraacetic acid (EDTA) solution (pH 7.8) for 8 weeks. After confirmation of decalcification on x-ray, knee joints were cut in halves along the medial collateral ligament in the frontal plane to directly get access to the central weightbearing region of the joint. The posterior half of the knees was dehydrated by transferring through solutions of increasing ethanol concentration up to 100% ethanol. After a final 24-hour dehydration step in cold 100% acetone at 4 °C, specimens were infiltrated with Technovit 8100 (VWR International) at 4 °C for 4 weeks. After this, specimens were placed into polyethylene-embedding molds. Polymerization solution (hardner), prepared according to the protocol of the manufacturer, was poured into the molds and air contact was prevented by covering the cavities with plastic films. The embedding form was placed on a thin layer of ice, and polymerization was allowed for 24 hours at 4 °C. After hardening was complete, specimens were blocked with Histobloc and Technovit 3040 (VWR International) and removed from the molds. Sections (5-10 µm) were cut from the blocks using a rotation microtome (Leica Biosystems, Nussloch, Germany), stretched on distilled water, and mounted on uncoated glass slides at 80 °C. Slides were subjected to thionine staining for routine histological examination by light microscopy (Axio Vert A1 microscope, Axiovision LE release 4.8.2; Carl Zeiss AG, Oberkochen, Germany).

As lesions in the ACLT/pMMx model develop mainly at the outer one-third of the medial tibial plateau, thionine-stained sections of the medial tibial plateau were scored using the cartilage degeneration score according to the OARSI histopathology initiative for the rat.^32^ Scoring was performed by 2 blinded observers (U.T.I. and R.M.J.). Measurements of parameters needed for the cartilage degeneration score were made using Axiovision Software (Axiovision LE release 4.8.2; Carl-Zeiss).

### Statistics and Data Analysis

#### ELISA and RNA measurements

GraphPad Prism 8 (GraphPad Software, San Diego, CA) was used for statistical analyses. Per donor, explants were obtained, pooled, and randomly divided over the different conditions. All samples for gene expression analysis and prostanoid measurements in the medium were processed and analyzed individually with a single measurement per donor. Continuous variables were tested for normality using the Kolmogorov-Smirnov test and normality plots were visually assessed for skewness. No normal distribution was identified. Effects of celecoxib on gene expression and prostanoid release by different intra-articular tissues were evaluated using a Wilcoxon matched-pairs signed-rank test. Statistical differences in histology scores in OA-induced knees or non-OA-induced knees injected with 0.9% NaCl or a bolus celecoxib were analyzed using a Mann-Whitney *U* test.

#### Proteome analysis

The acquired spectra were analyzed for protein identification and quantification using Proteome Discoverer (PD) Software version 2.2 (Thermo Fisher Scientific). Protein identification was conducted using the Sequest HT search engine with SwissProt (Human) database (Homo sapiens, Tax ID 9606). Analysis settings for this search included: enzyme trypsin, a maximum of 2 missed cleavage sites, a minimum peptide length of 6 and maximum of 144, a precursor mass tolerance of 10 ppm, and fragment mass tolerance of 0.02 Da. In addition, dynamic modifications of methionine oxidation (+15.995 Da) and protein *N*-terminus acetylation (+42.011 Da), and static modification of carbamidomethylation (+57.021 Da) were used. A false discovery rate of ≤1% was applied.

Protein quantification was performed using label-free quantification settings in PD version 2.2. Peptide precursor intensities were used for peptide abundance and total peptide amount was used for normalization. The difference in protein secretion (proteins with high confidence) between treatment groups was determined using a Wilcoxon signed-rank test with Benjamini-Hochberg correction for multiple testing^36^ in MATLAB 2018a for Windows (MathWorks, Natick, MA) with α = 0.05.

## Results

### Celecoxib Reduces Prostanoid Release by Cartilage *Ex Vivo*

**Figure 1.**
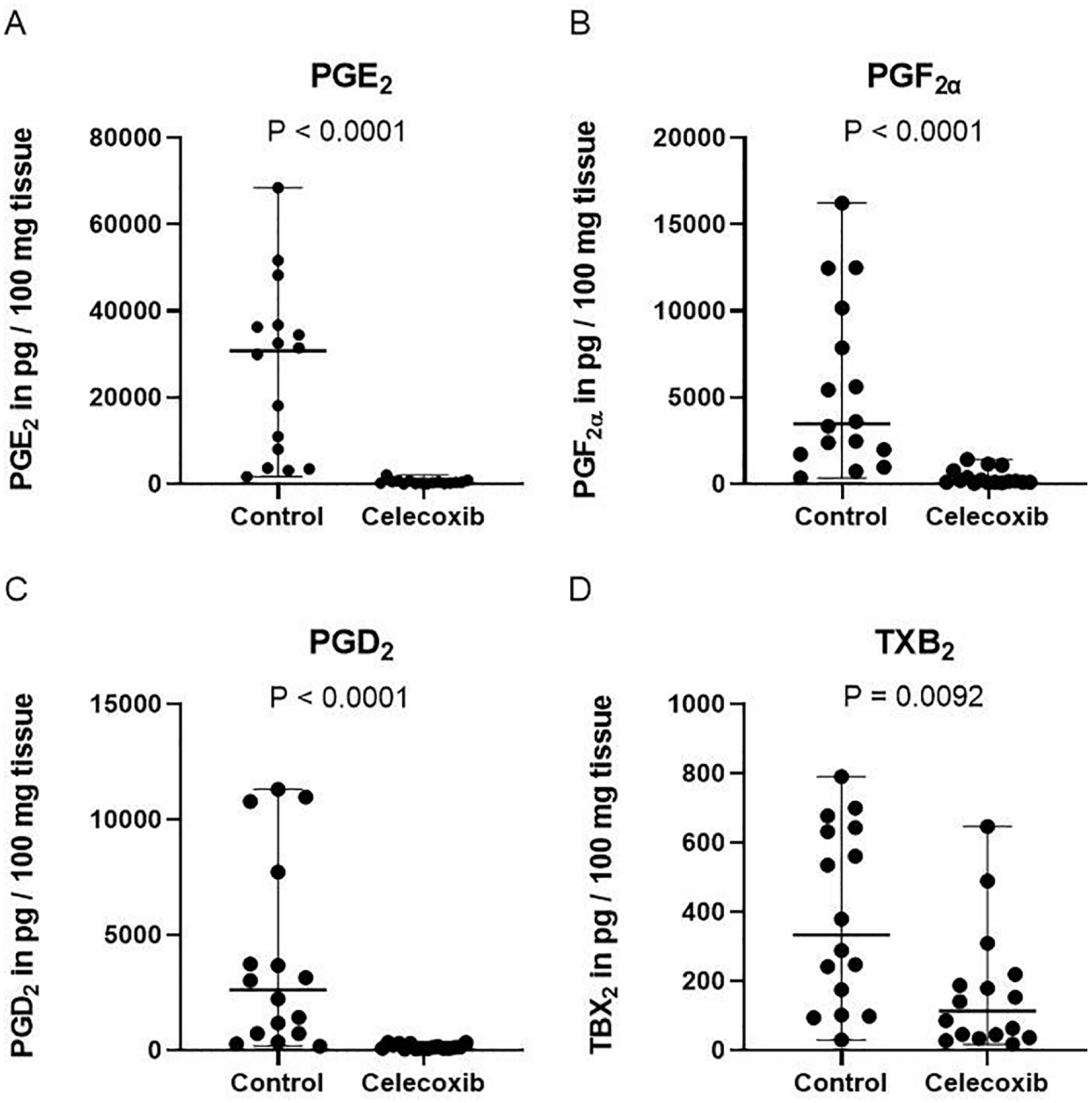
Celecoxib reduced prostanoid release by cartilage *ex vivo*. PGE2 (**A**), PGF2α (**B**), PGD2 (**C**) and TXB2 (**D**) release by cartilage after 24 hours of culture. Each dot represents absolute prostanoid values (in pg/100 mg tissue) per individual patient. The median and range are plotted in the figures. *P* values are depicted in the figure. *N* = 16. PGE_2_ = prostaglandin E_2_; PGD_2_ = prostaglandin D_2_; PGF_2α_ = prostaglandin F_2α_; TXB2 = thromboxane B2.

All 4 prostanoid subtypes were detected in medium conditioned by OA cartilage (**Fig. 1**). The average concentration of PGE_2_, PGF_2α_, PGD_2_, and TXB_2_ by cartilage in the control group was, respectively, 23 ng/ml, 5 ng/ml, 3 ng/ml, and 0.4 ng/ml (**Fig. 1**). In conditioned medium acquired from celecoxib-treated OA cartilage, a significantly reduced PGE_2_ (*P* < 0.001; 60-fold), PGF_2α_ (*P* < 0.001; 14-fold), PGD_2_ (*P* < 0.001; 21-fold), and TXB_2_ (*P* = 0.0092; 2-fold) concentration was measured (**Fig. 1**).

### Celecoxib Reduces Gene Expression of Proteolytic Enzymes in Cartilage *Ex Vivo*

**Figure 2.**
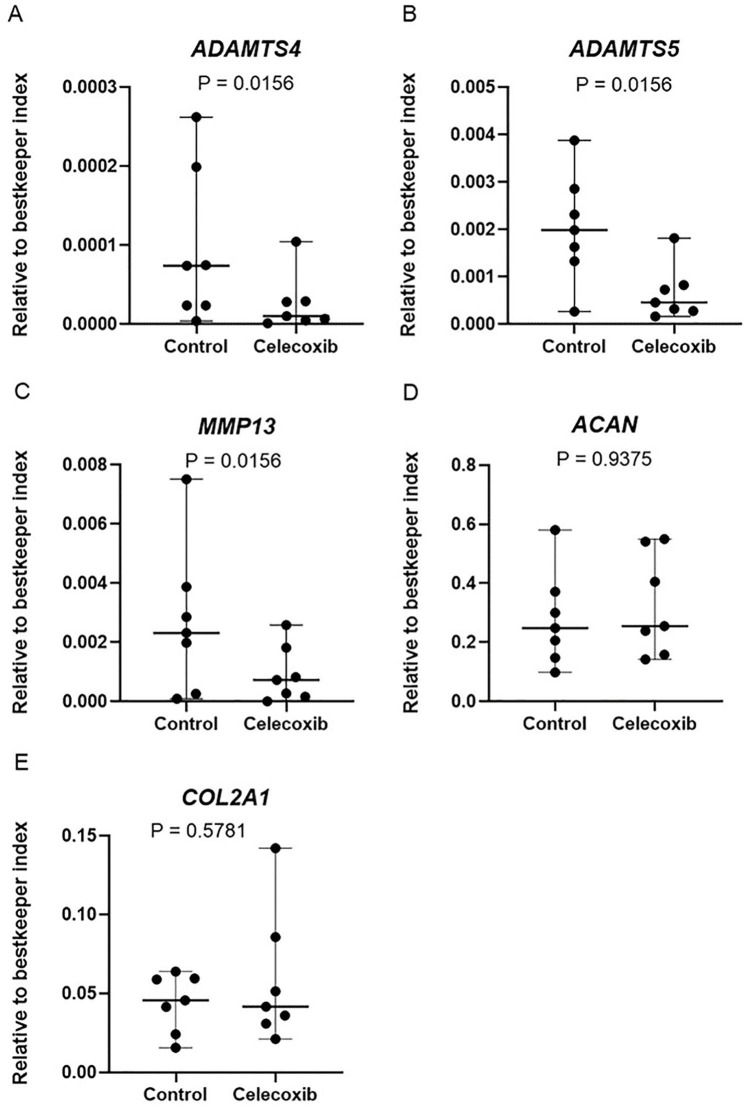
Celecoxib reduces gene expression of proteolytic enzymes in cartilage *ex vivo*. ADAMTS4 (**A**), ADAMTS5 (**B**), MMP13 (**C**), AGC (**D**), and COL2A1 (**E**) mRNA expression levels in cartilage explants from OA patients treated with or without celecoxib. The median and range are plotted in the figures. Absolute *P* values are depicted in the figure. *N* = 7. *ADAMTS4* = a disintegrin and metalloproteinase with thrombospondin motifs 4; *ADAMTS5* = a disintegrin and metalloproteinase with thrombospondin motifs 5; MMP13 = metalloproteinase 13; AGC = aggrecan (protein); COL2A1 = collagen type 2 alpha 1; OA = osteoarthritis.

To evaluate the chondroprotective effects of celecoxib on cartilage *ex vivo*, we evaluated the effect of celecoxib on the gene expression levels of *COL2A1* and *ACAN*, which are major structural proteins of the cartilage matrix.^37^ We have also evaluated the effect of celecoxib on the gene expression levels of proteolytic enzymes *MMP13, ADAMTS4*, and *ADAMTS5*, which are known to be involved in knee OA pathophysiology.^37^ The expression of *COL2A1, ACAN, MMP13, ADAMTS4*, and *ADAMTS5* could be detected in all cartilage donors. Celecoxib treatment did not alter the gene expression of *COL2A1* (*P* = 0.5781) and *ACAN* (*P* = 0.9375). However, it significantly reduced gene expression levels of *ADAMTS4* (*P* = 0.0156; 4-fold), *ADAMTS5* (*P* = 0.0156; 3-fold), and *MMP13* (*P* = 0.0156; 3-fold) in cartilage (**Fig. 2**).

### Celecoxib Changes Protein Secretion by Cartilage *Ex Vivo*

**Figure 3.**
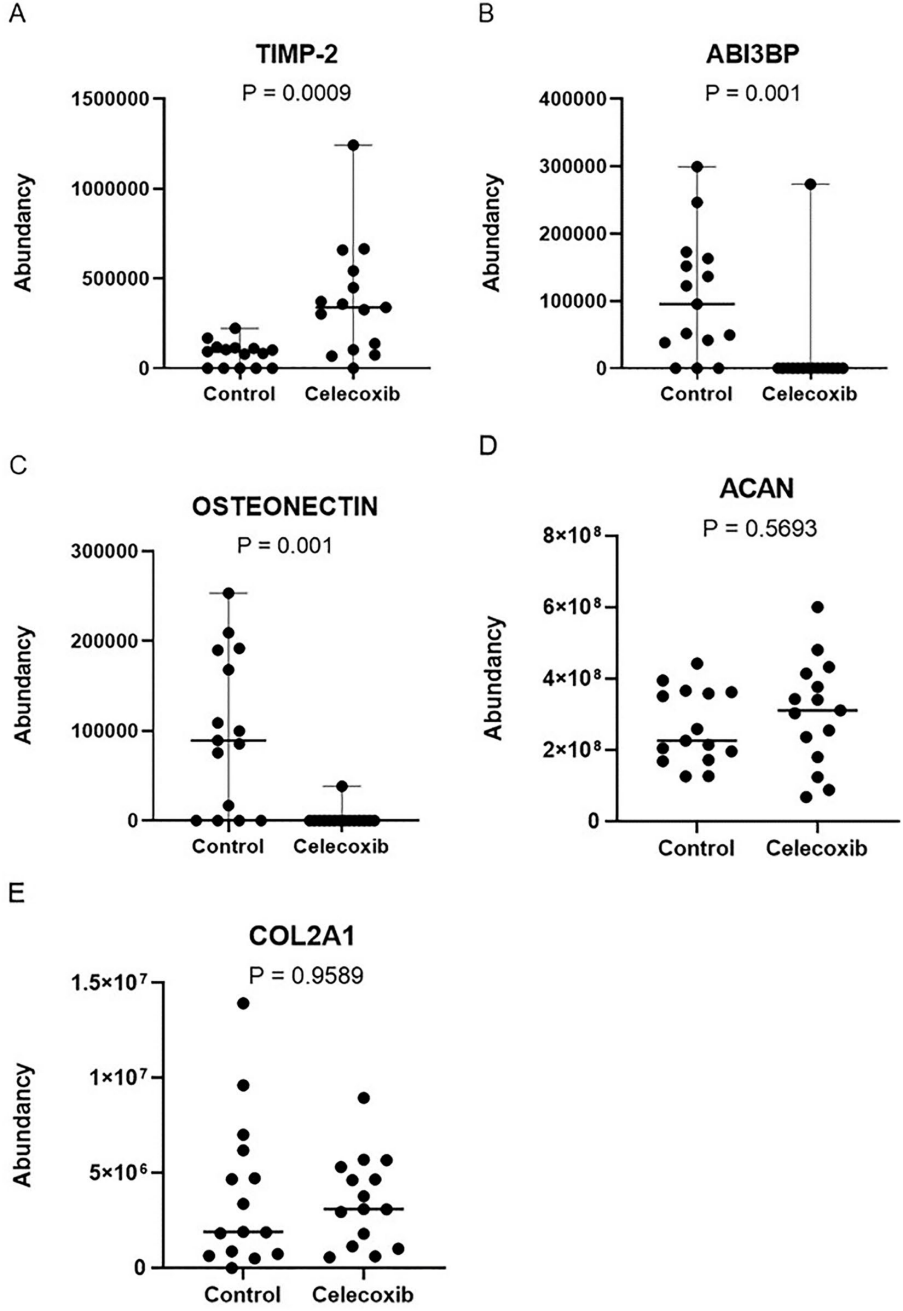
Significantly different secreted proteins by cartilage after celecoxib treatment. Changes in osteonectin (**A**), TIMP-2 (**B**), and ABI3BP (target of Nesh-SH3) (**C**) after celecoxib treatment. ACAN (**D**) and COL2A1 (**E**) did not change. The median and range are plotted in the figures. Absolute *P* values are depicted in the figure. *N* = 15. TIMP-2 = tissue inhibitor of metalloproteinase 2; ABI3BP = target of Nesh-SH3; ACAN = aggrecan; COL2A1 = collagen type 2 alpha 1.

Cartilage-conditioned media were analyzed using LC-MS/MS to get a broader insight into the actions of celecoxib on cartilage on secreted proteins. A total protein input of 6.6 μg/μl per sample was used for the detection of 154 proteins with high confidence in cartilage-conditioned media. COL2A1 and AGC core protein (aggrecan, *ACAN*) were detected in our analysis; however, there were no significant differences between groups (**Fig. 3**). A significant increase (after *P* value correction) in normalized abundance of tissue inhibitor of metalloproteinase 2 (TIMP-2; *P* = 0.0009; 5-fold) and reduction of target of Nesh-SH3 (ABI3BP; *P* = 0.0010; 6-fold) and osteonectin (*P* = 0.0010; 39-fold) was observed after treatment with celecoxib (**Fig. 3**).

### Celecoxib Acts Chondroprotective *In Vivo*

**Figure 4.**
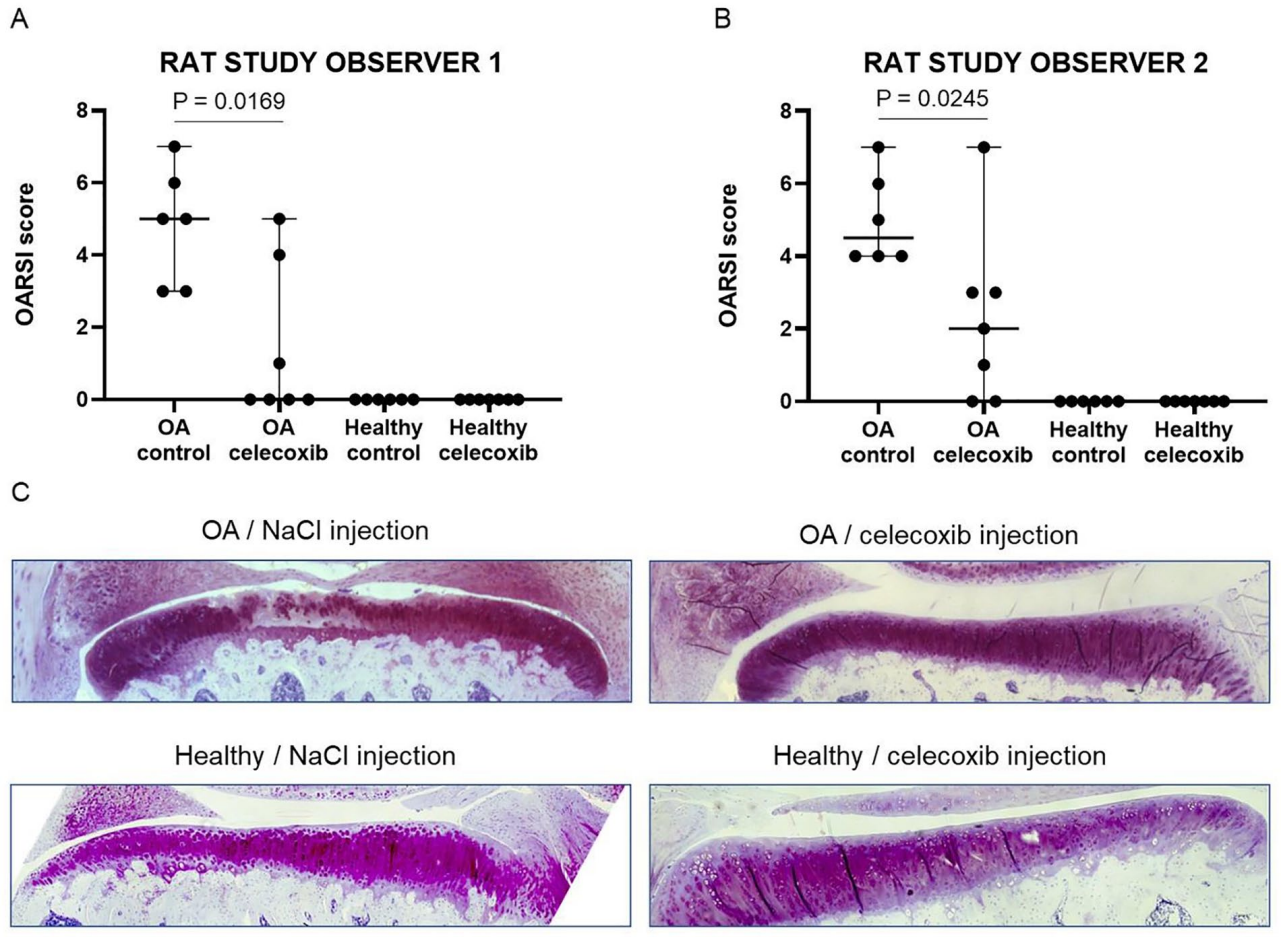
Cartilage degeneration scores of rat knees treated with celecoxib or NaCl. Cartilage degeneration scoring of rat knees by observer 1 (**A**) and observer 2 (**B**), including histological images representing the largest difference of a rat knee injected with NaCl or celecoxib (**C**). The median and range are plotted in figures A and B. Absolute *P* values are depicted in the figure. *N* = 6 for OA and healthy with NaCl treatment. *N* = 7 for OA and healthy celecoxib. OA = osteoarthritis.

Based on the *ex vivo* results showing the anti-catabolic effect of celecoxib, we investigated the effect of a single intra-articular bolus injection with celecoxib *in vivo* on OA development in a trauma-induced OA rat model (ACLT/pMMx model).^32^ No wound infection was noticed after ACLT and pMMx surgery in any of the animals. We had a dropout of one of the rats in the control group. The wounds healed within 1 week with no difference between the operated and non-operated leg. All rats had a similar weight gain over 16 weeks. The cartilage degeneration score^32^ was significantly reduced in OA-induced knees treated with celecoxib compared with OA-induced knees injected with 0.9% NaCl, as scored by observer 1 (median cartilage degeneration score: 0.9% NaCl 5 [range 3-7] and celecoxib 0 [range 0-5]; *P* = 0.0169; **Fig. 4**) and observer 2 (median cartilage degeneration score: 0.9% NaCl 4.5 [range 4-7] and celecoxib 2 [0-7]; *P* = 0.245; **Fig. 4**). Contralateral knees without OA induction did not have significant cartilage pathology in both the 0.9 % NaCl group and bolus celecoxib group (**Fig. 4B**). Histological images representing the difference between OA-induced and healthy rat knees injected with 0.9 % NaCl and a bolus celecoxib are shown in **Figure 4**.

## Discussion

As hypothesized in this study, cartilage prostanoid (PGE_2_, PGF_2α_, PGD_2_, and TXB_2_) levels in cartilage-conditioned medium were reduced after treatment with celecoxib. In addition, celecoxib altered gene expression levels of proteolytic enzymes in cartilage *ex vivo*, but did not alter gene expression of *COL2A1* and *ACAN*. Celecoxib did significantly reduce gene expression levels of *MMP13, ADAMTS4*, and *ADAMTS5* in cartilage, suggesting an anti-proteolytic effect. In addition, an untargeted LC-MS/MS analysis was conducted on medium samples of all explant cultures to confirm that the changes in gene expression led to different protein secretion profiles. Three proteins showed significant upregulation (TIMP-2) or downregulation (ABI3BP and osteonectin) in the conditioned medium treated with celecoxib. Finally, we found an OA-modulatory effect *in vivo*. The cartilage degeneration score was significantly reduced in OA-induced knees treated with celecoxib compared with OA-induced knees injected with 0.9% NaCl. Overall, in accordance with our hypothesis, the data indicate a chondroprotective role of celecoxib *ex vivo* and *in vivo*.

In this work, we combined analysis on the effect of celecoxib on cartilage on different levels: the secretion of prostaglandins and proteins, gene expression, and its effect *in vivo* in a OA rat model. Prostanoid-reducing actions of celecoxib on cartilage have been shown earlier.^38^ In addition, as the specific COX-2 inhibitor celecoxib was able to significantly reduce prostanoid release in cartilage, prostanoid synthesis in cartilage seems to be mainly COX-2-driven. These data are in accordance with a study performed by Hardy *et al*.,^8^ where COX-2 induction was detected in cartilage after an inflammatory stimulus. We and others have also found that prostanoid subtypes differentially influence chondrogenic differentiation, indicating that prostanoids have an anti-catabolic function, and influence the chondrogenic differentiation of progenitor cells.^39,40^ The detection of all prostanoid subtypes in cartilage and their inhibition by celecoxib suggest that they are all COX-2-driven. Future experiments can focus on whether specific subtypes have different functions in cartilage and may aid in developing novel therapeutic strategies to target inflammatory and catabolic processes in knee OA.

In the current work, we provide further evidence of the chondroprotective effects of celecoxib via the modulation of other important proteolytic enzymes involved in knee OA such as *ADAMTS4* and *ADAMTS5*.^41^ *ADAMTS4* and *ADAMTS5* have been shown to play an important role in OA development by the degradation of *ACAN* (a critical cartilage component).^42^ In addition, MMPs (especially MMP13)^43^ contribute to this process by degrading collagen.^42,44^ Yang *et al*.^45^ found that celecoxib can reduce the expression of gelatinases in different joint tissues such as cartilage. These enzymes are involved in knee OA pathophysiology.^45^ The decrease of secretion of these enzymes by cartilage after celecoxib treatment suggests that celecoxib acts anti-proteolytic by regulation of the expression of these enzymes. In addition, it has been shown that celecoxib inhibits the production of MMPs via nuclear factor-κB and mitogen-activated protein kinases, unrelated to PGE_2_.^46^

In our study, celecoxib increased the secretion of TIMP-2. According to these results and given literature,^43,47,48^ this suggests the confirmation that celecoxib treatment might also lead to a decrease in MMP activity, inhibiting cartilage degradation and inflammation.^43,49^ Although a direct connection between TIMP-2 and *MMP-13* was not made in this experimental setting, the data support the indications that celecoxib may act anti-proteolytic and thus contribute to the chondroprotective effect on cartilage.

The untargeted LC-MS/MS analysis revealed a reduction in ABI3BP, an extracellular matrix structural constituent that has been associated with inducing cell senescence in different cell types.^50^ Senescence has been associated with OA,^51,52^ although little is known about the mechanisms involved in chondrocytes. ABI3BP might be one of the factors promoting cellular senescence in articular chondrocytes,^53^ and its downregulation in articular cartilage supports the potential chondroprotective effect of celecoxib. Similarly, osteonectin (secreted protein acidic and rich in cysteine [SPARC]) is an extracellular matrix protein, playing an important role in collagen binding and modulating cell-matrix interactions.^54,55^ Osteonectin binds calcium and is involved in cartilage calcification, leading to the progression of OA.^56^ Its downregulation after treatment with celecoxib further bolsters insights into a potential chondroprotective effect of celecoxib.

In the *in vivo* rat model for OA, we used one single bolus injection of celecoxib to ensure only local administration, in line with our previous data.^19^ Previously performed animal studies^18,25-27^ using intra-articular injection of celecoxib show contradictive results, dependent on dosage and way of administration (drug delivery system or single bolus injection), initiation, and duration of treatment and scoring system. In our study, we do not make use of any drug delivery system or repeated intra-articular injections. In addition, our study initiates celecoxib treatment at 4 weeks after surgery, suggesting that OA was developed further than when started directly after OA induction by surgery. Although our data imply a positive outlook for the use of celecoxib as chondroprotective drug in OA patients, the discussion remains whether this contrasting evidence is due to factors such as celecoxib concentration, timing of injection, or the fact that celecoxib might function analgesic, leading to improved mobility (and increased cartilage wear and OA development) of the treated animal.^24^ Overall, according to our results, we speculate that celecoxib might cause an effective reduction of local inflammation and that this might be accompanied by an anti-proteolytic effect downstream of the inflammatory signaling.

As our results suggest that celecoxib can be used as a chondroprotective drug in the treatment of osteoarthritis, it should be noted that this only includes the beneficial effects and that certain side effects, ranging from gastrointestinal problems to cardiovascular diseases and high blood pressure, should be taken into account.^20,57^ In addition, we hypothesize for future studies that local administration of celecoxib, by intra-articular injection, might not only have a positive influence on its chondroprotective effect, but might also limit certain systemic side effects that have been seen with the use of celecoxib.^20^ With the application of local administration, it could be easily regulated how much celecoxib eventually ends up at the affected location in the joint, rather than the rest of the body. With this, we reopen the discussion and try to close the knowledge gap on the best way for celecoxib to be administrated.

One limitation of our study is the use of a rodent animal model. Nevertheless, we were still able to conclude that celecoxib acts chondroprotective *in vivo* when administrated locally using a single intra-articular injection. Future studies should focus on bigger animal models such as goat, sheep, or horse, in general more similar to humans. Our data suggest the importance of implementing local drug administration for osteoarthritis treatment.

In conclusion, our data indicate a chondroprotective effect of celecoxib via a reduction in inflammation and proteolytic enzyme expression. A single bolus injection of celecoxib showed a protective effect against cartilage degeneration in a rat model for OA. These data suggest that local, intra-articular administration of celecoxib might be more effective in OA treatment and that celecoxib should be reconsidered as a chondroprotective drug in OA patients. However, certain side effects should be taken into account when prescribing this drug. Future experiments should therefore focus on local administration and gathering more *in vivo* data, especially from bigger animal models and human studies.

## Supplemental Material

sj-docx-1-car-10.1177_19476035221115541 – Supplemental material for Evaluation of the Anti-Inflammatory and Chondroprotective Effect of Celecoxib on Cartilage Ex Vivo and in a Rat Osteoarthritis Model Click here for additional data file. Supplemental material, sj-docx-1-car-10.1177_19476035221115541 for Evaluation of the Anti-Inflammatory and Chondroprotective Effect of Celecoxib on Cartilage Ex Vivo and in a Rat Osteoarthritis Model by Mirella J.J. Haartmans, Ufuk Tan Timur, Kaj S. Emanuel, Marjolein M.J. Caron, Ralph M. Jeuken, Tim J.M. Welting, Gerjo J.V.M. van Osch, Ron M.A. Heeren, Berta Cillero-Pastor and Pieter J. Emans in CARTILAGE
