# Supplementary material for: Evaluation of the Anti-Inflammatory and Chondroprotective Effect of Celecoxib on Cartilage Ex Vivo and in a Rat Osteoarthritis Model
Source: Cartilage. 2022 Aug 5;13(3):19476035221115541. doi: 10.1177/19476035221115541 (PMC9364198; doi:10.1177/19476035221115541)
Supplement: sj-docx-1-car-10.1177_19476035221115541 – Supplemental material for Evaluation of the Anti-Inflammatory and Chondroprotective Effect of Celecoxib on Cartilage Ex Vivo and in a Rat Osteoarthritis Model [file sj-docx-1-car-10.1177_19476035221115541.docx]

**List of abbreviations**

ABI3BP Target of Nesh-SH3

ACAN Aggrecan (gene)

ACL Anterior cruciate ligament

ACLT/pMMx Anterior cruciate ligament transection/partial medial meniscectomy

ACN Acetonitrile

ADAMTS4 A disintegrin and metalloproteinase with thrombospondin motifs 4

ADAMTS5 A disintegrin and metalloproteinase with thrombospondin motifs 5

AGC Aggrecan (protein)

COL2A1 Collagen type 2 alpha 1

COX Cyclooxygenase

DDA Data-dependent acquisition

DMEM Dulbecco’s Modified Eagle Medium

DMSO Dimethylsulfoxide

DOC Sodium deoxycholate

ECM Extracellular matrix

ELISA Enzyme-Linked Immunosorbent Assay

FA Formic acid

FDR False discovery rate

GAPDH Glyceraldehyde 3-phosphate dehydrogenase

ITS Insulin-transferrin-selenite

LC-MS Liquid chromatography-mass spectrometry

LC-MS/MS Liquid chromatography-tandem mass spectrometry

MMP13 Matrix metalloproteinase 13

MS Mass spectrometry

MS/MS Tandem mass spectrometry

NSAID Nonsteroidal anti-inflammatory drug

OA Osteoarthritis

PCA Principal component analysis

PD Proteome Discoverer

PG Prostaglandin

PPIA Peptidylprolyl isomerase A

TCA Trichloroacetic acid

TIMP-2 Metalloproteinase inhibitor 2

TKA Total knee arthroplasty

TX Thromboxane
